# The Role of Plectin Dysregulation in Cancer: Recent Advances

**DOI:** 10.3390/molecules30183675

**Published:** 2025-09-10

**Authors:** Wenbin Wang, Chang Lyu, Zhihui Wang, Xu Zhang, Qing Luo, Guanbin Song

**Affiliations:** 1 College of Bioengineering, Chongqing University, Chongqing 400030, China; 202319021096t@stu.cqu.edu.cn (W.W.); 20233265@stu.cqu.edu.cn (C.L.); 202219021037@stu.cqu.edu.cn (Z.W.); 202419021021@stu.cqu.edu.cn (X.Z.); qing.luo@cqu.edu.cn (Q.L.); 2 Key Laboratory of Biorheological Science & Technology, Ministry of Education, Chongqing University, Chongqing 400030, China

**Keywords:** plectin, dysregulation, tumor growth, invasion and metastasis, tumor microenvironment, cancer therapy

## Abstract

Plectin is a key cytolinker protein that functions as an integrator of the cytoskeletal networks by crosslinking intermediate filaments with actin filaments and microtubules. Mutations or function deficiencies of plectin lead to tissue disorders, particularly affecting the skin, muscle, and nervous tissues. Interestingly, plectin dysregulation in cancer, characterized by aberrant expression and mislocalization, has been increasingly observed, suggesting distinct roles in tumorigenesis and progression. Here, we focus on recent advances regarding the roles of plectin dysregulation in promoting cell proliferation, suppressing cell apoptosis, sustaining the stemness of cancer stem cells, and driving invasion and metastasis. We also discuss its bidirectional interplay with the tumor microenvironment, including modulating immune and inflammatory responses, promoting angiogenesis, sensing and transmitting mechanical cues from the extracellular matrix, and contributing to matrix remodeling. Finally, we highlight emerging therapeutic strategies that target plectin dysregulation with anticancer activity. By summarizing these advances, we aim to enhance the understanding of plectin dysregulation in cancer and illuminate its potential as a therapeutic target.

## 1. Introduction

Plectin is a 500 kDa cytolinker protein encoded by the *PLEC* gene, also known as plectin-1 [1,2] or hemidesmosome protein 1 [3,4]. Structurally, it comprises an N-terminal actin-binding domain (ABD), a plakin domain, a central rod domain, and a C-terminal plectin repeat domain [5]. Through these domains, plectin associates with various components of the cytoskeleton, including actin filaments (AFs), intermediate filaments (IFs), and microtubules [6]. In addition, plectin participates in the assembly of cell junctions such as desmosomes, hemidesmosomes (HDs), and focal adhesions (FAs) [7,8,9,10,11]. The human *PLEC* gene coding sequence consists of 32 exons and spans approximately 32 kb, located in the telomeric region of chromosome 8 (8q24) [12]. Notably, the *PLEC* gene generates multiple isoforms through the alternative splicing of the 5′-terminal first exons, among which plectin 1, 1a, 1b, 1c, 1d, 1f, and 1k are the most extensively characterized [5,13,14]. Plectin 1 localizes to the perinuclear region [15]; plectin 1a anchors to hemidesmosomes [7]; plectin 1b regulates mitochondrial positioning and morphology [16]; plectin 1c associates with microtubules [7]; plectin 1d links the desmin intermediate filament network to the Z-disks of muscle fibers [17]; plectin 1f is enriched at focal adhesions [11]; and plectin 1k is involved in F-actin organization and podosome-like adhesions formation [18]. These isoforms primarily influence the binding properties of the ABD, resulting in structural specificity and targeting distinct cellular structures [14,19].

Under normal physiological conditions, plectin is predominantly localized in the cytoplasm, where it anchors cytoskeletal components and maintains the cell’s structural integrity [20]. Mutations in the *PLEC* gene or plectin deficiencies have been linked to a spectrum of human diseases, characterized by muscle weakness and atrophy, skin fragility and blistering, as well as signs of neuropathy [21,22]. Interestingly, emerging evidence in recent years has revealed the dysregulation of plectin in cancer. According to the ground-breaking discovery in 2008, plectin was first identified as a cell surface biomarker for pancreatic ductal adenocarcinoma (PDAC), despite its conventional intracellular localization [23]. Since then, accumulating evidence has demonstrated that both the expression levels and subcellular localization of plectin are frequently dysregulated in cancer [24,25,26,27,28]. Its mislocalization has been shown to confer unexpected functions in cancer progression, including the promotion of tumor growth and metastasis, as well as the maintenance of cancer stem cells (CSCs) stemness. Notably, the form of plectin that is aberrantly localized on the surface of tumor cells and exhibits pro-tumorigenic functions is referred to as cancer-specific plectin (CSP) [23,25]. The discovery of CSP underscores the pathological mislocalization of plectin and provides a promising avenue for tumor detection and targeted therapy.

Plectin dysregulation has emerged as a critical driver of tumor progression. It promotes tumor growth by sustaining cell proliferation [29], inhibiting apoptosis [30], and supporting the stemness of CSCs [27]. In addition, plectin functions as a metastasis-promoting factor by modulating signaling pathways involved in cell migration and invasion [31]. Recent evidence reveals that the dysregulated expression of plectin modulates the cellular and non-cellular architecture of the tumor microenvironment (TME), thereby facilitating a supportive niche for tumor invasion and dissemination. In cancer cells, plectin enhances resistance to mechanical stress [32] and is associated with hypoxic conditions [33]. Moreover, plectin contributes to immune evasion [34], extracellular matrix degradation [26], activation of inflammatory signaling [35], and angiogenesis [36], collectively promoting the establishment of a permissive pre-metastatic niche. In this review, we summarize the oncogenic roles of plectin dysregulation in tumor growth, metastasis, and its bidirectional interplay with the TME. We also discuss the novel therapeutic strategies targeting plectin dysregulation to promote precision cancer treatment, including small molecules, mRNA therapies, and antibody therapies.

## 2. Dysregulation of Plectin in Cancer

Although plectin deficiency is known to cause tissue disorders, a clearer understanding of plectin dysregulation and its implications in cancer is still necessary. In malignancies, the *PLEC* gene frequently undergoes copy number variations, leading to aberrant plectin expression, which is increasingly recognized as a potential hallmark of malignancy. More importantly, plectin in cancer cells often exhibits alterations in subcellular localization, shifting from its typical cytoplasmic distribution to the cell membrane. Here, we summarize the dysregulation of plectin, including its upregulation, downregulation, and mislocalization in different types of cancers, as shown in Table 1.

### 2.1. Upregulation of Plectin in Cancer

Plectin is primarily upregulated in cancer and contributes to tumorigenesis and tumor development. It is notably elevated in nearly all gastrointestinal cancers, even in precancerous lesions. For example, plectin expression is upregulated in pancreatic intraepithelial neoplasms (PanINs) and intraductal papillary mucinous neoplasms compared to normal pancreatic tissue [23,60]. This suggests that the upregulation of plectin may play a crucial role in the early stages of tumorigenesis. Similarly, abnormal overexpression of plectin has been reported in reproductive cancers, such as prostate cancer, testicular cancer, and ovarian cancer. In prostate cancer, relative to benign tissues, plectin expression is increased in both primary tumors and lymph node metastases [30], indicating a potential role in metastatic progression. Elevated plectin levels have also been documented in head and neck cancers [50]. Plectin has been proposed as a potential biomarker for early lesion detection and disease progression in oral squamous cell carcinoma (OSCC) [28]. Beyond these cancer types, aberrant plectin expression is observed in other malignancies. For example, an abnormal enrichment of plectin-positive spindle stromal cells has been observed in the bone marrow of acute myeloid leukemia (AML) patients [37]; glioblastoma multiforme (GBM) cell lines exhibit higher levels of surface plectin microdomains compared to normal astrocytes [48]; and plectin is upregulated in human primary melanoma samples relative to normal melanocytes [29].

Upregulation of the *PLEC* gene expression has been associated with cancer patient prognosis. In OSCC, lower *PLEC* gene expression is linked to a favorable prognosis, particularly among patients without lymph node metastasis and those with T1-stage tumors [28]. In GBM, both *PLEC* gene expression and mutation status have been proposed as predictive biomarkers for the efficacy of ADP-ribose polymerase inhibitors and patient outcomes [68]. In PTEN-negative prostate cancer, elevated *PLEC* gene expression serves as an independent prognostic factor, correlating with more aggressive disease and poorer clinical outcomes [61]. Similarly, in the TCGA bladder cancer cohort, high *PLEC* gene levels correlate with decreased overall and disease-free survival in patients with bladder cancer [69]. However, the prognostic significance of the *PLEC* gene appears to be specific to cancer type. For instance, in ovarian cancer, no significant correlation has been observed between *PLEC* gene expression and overall or progression-free survival [56], highlighting the heterogeneous role of plectin in tumor biology.

### 2.2. Downregulation of Plectin in Cancer

Although plectin is upregulated in most tumor types, its downregulation has also been observed in certain malignancies, indicating a dual expression pattern during cancer progression. For example, in basal and squamous cell carcinomas, plectin is expressed at lower levels in tumor tissues compared to normal skin tissue as a component of HDs [64]. Interestingly, even within the same tumor type, contradictory reports have emerged. In esophageal squamous cell carcinoma (ESCC), plectin is frequently overexpressed. However, its knockdown has been shown to promote carcinogenesis by disrupting the homeostasis of the stratified squamous epithelium (SSE), indicating that plectin downregulation may also contribute to tumor progression [45].

In hepatocellular carcinoma (HCC), the most robust evidence comes from Outla et al. (2025), who reported elevated plectin expression based on 17 independent patient datasets and quantitative immunofluorescence analysis of 19 paired tissue sections [26]. Functional studies demonstrated that inhibition of plectin significantly suppressed HCC proliferation, migration, invasion, cytoskeletal remodeling, and tumor growth via CRISPR/Cas9-mediated knockout, deletion of the intermediate filament-binding domain, or pharmacologic inhibition with plecstatin-1 (PST) in Huh7 and SNU-475 cells [26]. Xu et al. (2022) similarly found that shRNA-mediated plectin knockdown in MHCC97H and MHCC97L reduced extracellular signal-regulated kinase 1/2 (ERK1/2) phosphorylation, inhibiting cell migration and the epithelial–mesenchymal transition (EMT) process [31]. Furthermore, Wang et al. (2025) demonstrated that depletion of either plectin or integrin β1, or disruption of actin polymerization with latrunculin A, effectively blocked mechanical stiffness-induced F-actin polymerization and HCC cell migration [32].

Conversely, earlier studies have reported weaker plectin expression in HCC. Cheng et al. (2008) [70] observed strong plectin staining in normal liver tissue but weaker staining in tumor regions. This pattern was further supported by immunohistochemical analysis of five HCC cases, which showed markedly reduced plectin expression in tumor regions [70]. Similarly, Liu et al. (2011) observed weaker plectin staining in HCC sections compared with adjacent non-tumorous tissue across ten cases, consistent with Western blot results [71]. Additionally, Cheng (2015) noted heterogeneous morphology and relatively weak plectin staining in tumor regions of HCC patient tissues [65].

Notably, plectin expression in ovarian cancer appears to follow a dynamic pattern: it is upregulated during early tumorigenesis but subsequently downregulated as the disease progresses to high-grade or advanced stages [56]. Collectively, these findings highlight the complex and dual roles of plectin in cancer, indicating that its expression and function may vary among tumor types, stages, and histological subtypes.

### 2.3. Mislocalization of Plectin in Cancer

Plectin was initially characterized as a cytoplasmic protein ubiquitously expressed across mammalian tissues [13,24]. However, accumulating evidence suggests that plectin frequently translocates to the plasma membrane in cancer cells. It was first identified as a membrane-associated biomarker in PDAC, with cell surface expression progressively increasing from PanIN III to invasive PDAC [23]. Subsequently, an unbiased peptide-binding screen revealed plectin as a unique membrane marker for ALDH^+^ lung cancer stem cells [27]. Recent studies further demonstrated that in HCC, both clinical samples and chemically induced mouse models exhibit significant perimembranous enrichment of plectin [26]. In colorectal cancer (CRC), Ras-related protein Rap-2B (Rap2B) was found to colocalize with plectin in both the cytoplasm and plasma membrane. Rap2B translocates to the membrane via its C-terminal CAAX motif (C180), where it directly binds to the ABD of plectin, thereby promoting their membrane co-localization [72]. In ovarian cancer, a novel monoclonal antibody (1H11) was developed to specifically recognize CSP on the surface of both human and mouse tumor cells, confirming its membrane localization [25].

Plectin isoforms regulate the subtype-specific distribution of cytoskeletal connections and organelle localization through their variable N-terminal sequences. Consequently, the aberrant subcellular localization of plectin in cancer cells may result from the dysregulation of specific isoforms. In GBM, plectin 1c is the predominant isoform and shows notable co-localization with plasma membrane-associated aquaporin 4 [48]. In addition, plectin 1c is involved in HD composition, thus exhibiting peripheral localization [5]. In CRC, plectin 1 and plectin 1k are upregulated and specifically enriched at podosome-like adhesions in highly invasive cells, supporting their localization at membrane-associated adhesions [18]. In PDAC, the plectin isoforms 1a and 1f are abnormally localized on the cell surface via an exosome-mediated mechanism [24]. These findings highlight the crucial role of isoform-specific expression in regulating plectin localization and function during cancer progression.

## 3. The Role of Plectin Dysregulation in Cancer

**Figure 1:**
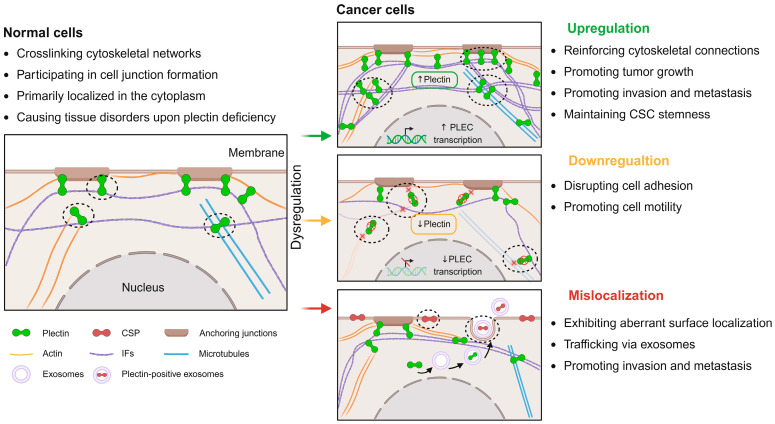
The impact of plectin dysregulation in cancer. In normal cells, plectin acts as a vital cytoskeletal linker protein and part of anchoring junctions, collectively maintaining cytoskeletal organization. However, plectin is dysregulated, exerting multifaceted effects on tumor progression. Its upregulation (indicated by upward arrows) facilitates cytoskeletal network crosslinking and activates signaling pathways that drive cancer cell growth, invasion, metastasis, and maintenance of cancer stemness. Conversely, decreased plectin expression (indicated by downward arrows) disrupts cell junctions and cytoskeletal architecture (indicated by cross signs), increasing cancer cell motility and morphological heterogeneity. Additionally, plectin mislocalizes to the cell membrane in cancer cells and may be trafficked via exosome formation. This process further regulates tumor growth and migration. Image created with BioRender.com.

Beyond its structural role in normal tissues, the dysexpression and mislocalization of plectin have been widely acknowledged for their functional relevance in cancer. Studies have shown that plectin dysregulation contributes to tumor progression through multiple mechanisms, such as promoting tumor growth, enhancing metastatic potential, and contributing to therapy resistance. In addition, plectin has increasingly been implicated as a key mediator of tumor–stroma interactions, contributing to the resistance and remodeling of the TME and fostering a pro-tumorigenic niche (As shown in Figure 1).

### 3.1. Sustaining Tumor Growth

Tumor growth is governed by a complex interplay of cellular processes, including survival, apoptosis, proliferation, and dormancy [73]. Among these hallmarks, sustained proliferation is a key feature of cancer progression, in which plectin has emerged as an essential regulator. Downregulation of plectin reduces tumor mass and attenuates cell proliferation by inhibiting the activity of the Src oncogene in melanoma cells [29]. In breast cancer, the oncogenic protein FAM83A interacts with plectin, modulating cytoskeletal architecture and activating downstream signaling pathways that promote cell survival and proliferation [74]. Additionally, plectin directly influences the expression of cell cycle regulators—for instance, silencing of plectin results in reduced expression of Cyclin D1 [29]. In the esophageal SSE, plectin connects HDs, desmosomes, and cytoskeletal elements to Notch1 and the *PLEC* gene regulator p63, playing a crucial role in maintaining cell anchorage, proliferation-differentiation equilibrium, and stratification. Thus, its dysregulation and mislocalization of plectin disrupt SSE homeostasis and contribute to ESCC carcinogenesis [45]. Collectively, these findings highlight the crucial role of plectin dysregulation in sustaining proliferative signaling essential for tumor growth.

Secondly, plectin contributes to resistance to apoptosis in cancer. It serves as a key target for caspase-mediated cytoskeletal disassembly during programmed cell death, with its cleavage facilitating apoptosis by disrupting the cytoskeletal framework. Plectin has been identified as a substrate of caspase-8 in both CD95- and tumor necrosis factor receptor-mediated apoptotic pathways, leading to the destabilization of the actin cytoskeleton [41]. Subsequent studies have demonstrated that plectin is also cleaved by caspase-3 and caspase-7, and this cleavage is blocked by the pan-caspase inhibitor zVAD-fmk, confirming the caspase-dependent nature of this process [75]. Clusterin, a glycoprotein that functions as a small heat shock protein, has been shown to induce apoptosis when translocated to the nucleus [76]. Preliminary findings suggest that plectin knockdown is associated with cell cycle arrest mediated by nuclear translocation of clusterin [30]. These findings indicate that plectin upregulation in cancer cells may help preserve cytoskeletal integrity and suppress apoptosis, thereby facilitating tumor cell survival.

Thirdly, the mislocalization of plectin has been identified as a crucial factor in promoting tumor growth. In PDAC, plectin is recruited into exosomes through an integrin β4-dependent pathway, resulting in its aberrant redistribution to the cell surface. These plectin-positive exosomes can enhance tumor growth even in recipient cells that lack plectin expression on their surface. Notably, the inhibition of exosome secretion via the knockout of Ras-related protein Rab-27A significantly impairs tumor growth, while supplementation with plectin-positive exosomes reverses this effect [24]. These findings underscore the tumor-promoting role of plectin-positive exosomes. In CRC, the membrane localization of plectin is modulated by Rap-2B, which in turn regulates F-actin dynamics and supports cellular proliferation [72]. Collectively, these observations demonstrate the functional significance of plectin mislocalization in promoting tumor progression.

### 3.2. Promoting Invasion and Metastasis

Cancer metastasis is the most advanced and lethal stage of cancer progression [77]. During EMT, the cytoskeleton undergoes extensive reorganization, which is closely associated with the invasive and metastatic potential of cancer cells. Plectin is frequently upregulated in metastatic tumors [78], while its downregulation inhibits cancer cell migration through various mechanisms. Structurally, plectin anchors intermediate filaments to cell–cell and cell–matrix junctions, such as vimentin, which can be recruited to focal adhesions via plectin isoform 1f, facilitating direct interactions with integrins [79]. Loss of plectin disrupts cytoskeletal integrity [26], shortens invadopodia [44], diminishes adhesion stability [45], and disrupts the integrin β4–plectin complex, thereby reducing metastatic potential [49].

Through this cytoskeletal anchoring, plectin maintains basal cell–ECM attachment and regulates focal adhesion kinase (FAK) activity, Src, ERK1/2, and p38 signaling, as well as RhoA-dependent actomyosin contractility [11]. Disruption of this linkage perturbs intracellular tension and adhesion dynamics, potentially compromising the mechanical feedback required for coordinated cell migration. Moreover, plectin promotes metastasis through Rap2B-dependent inhibition of F-actin polymerization, thereby altering cytoskeletal dynamics [72]. These findings highlight the role of plectin as an integrator of the mechanical network, influencing cancer cell motility and potentially regulating EMT-associated signaling proteins.

Beyond its structural roles, plectin integrates these structural and mechanical cues with signaling pathways to drive tumor growth and metastasis. In HCC, plectin is upregulated and promotes tumor cell motility through the activation of EMT and ERK1/2 signaling [31], whereas silencing plectin in HNSCC or HCC significantly inhibits migration and invasion [26,50]. In breast cancer, siRNA-mediated depletion of plectin suppresses migration, invasion, and adhesion, attenuating tumor growth and metastasis via the NF-κB1/CXCL9 axis [38]. Collectively, these studies highlight plectin as a central integrator of cytoskeletal remodeling, mechanotransduction, and the EMT process, coordinating intracellular tension, adhesion dynamics, and mesenchymal plasticity to drive metastatic dissemination across diverse tumor types.

Nevertheless, in the absence of plectin, human hepatocytes demonstrate higher motility and increased FAK activity, comparable to the invasive characteristics seen in infiltrative HCC [65]. This downregulation may weaken cell adhesion by impairing the formation and stability of HDs, thereby facilitating invasion and metastasis [80,81]. Within HDs, plectin forms a stable adhesion complex with type XVII collagen (Bullous pemphigoid antigen 180) and the integrin α6β4, which is essential for maintaining the balance between cell adhesion and motility in tumor cells [82,83]. Activation of the receptor disrupts the plectin–integrin β4 interaction through a PI3K-dependent mechanism, promoting pancreatic cancer cell migration [84]. In PTEN-deficient cells, disruption of α6β4 integrin-mediated HDs leads to plectin re-localization from the IFs network to integrin-rich focal adhesions, where it co-localizes with focal adhesion proteins such as paxillin and activates FAK/Src and EGFR/PI3K/Akt signaling pathways [61,65]. Moreover, in epithelial ovarian cancer, high plectin expression has been shown to suppress EMT-associated migration by interfering with membrane receptor-mediated signaling [56].

The aberrant localization of plectin on the cell surface is closely associated with enhanced invasive and metastatic potential in cancer cells. The plectin 1k isoform, which localizes to podosome-like structures, promotes cell migration and invasion by regulating AFs organization, integrin-mediated signaling, and cytoskeletal coordination. Notably, exogenous overexpression of plectin 1k effectively rescues the migratory deficits caused by endogenous plectin depletion [18]. Plectin can also be packaged into exosomes via integrin β4-dependent recruitment and transferred to plectin-negative recipient cells, where it localizes to the membrane and markedly enhances their motility and invasiveness [24]. Membrane-associated isoforms plectin 1a and plectin 1f, similarly released through exosome transport, have been shown to drive pancreatic tumor progression toward a more invasive phenotype [24]. Moreover, SNRPA1-mediated alternative splicing of exon 31 produces gliadin-like plectin isoforms with pro-metastatic properties, promoting lung colonization in breast cancer by modulating focal adhesions [42]. These findings indicate that plectin isoforms normally localized at cell–cell junctions may contribute to surface translocation in cancer cells, thereby facilitating migration, invasion, and metastatic dissemination.

### 3.3. Sustaining Tumor Stemness

CSCs are a rare subpopulation within the tumor tissues with the capacity of self-renewal and differentiation [85]. They are considered major contributors to metastasis and therapy resistance, posing a critical challenge to effective cancer treatment [86]. Emerging evidence suggests that plectin functions as a potential regulator of CSC properties [87]. Specifically, plectin has been identified as a novel surface marker for ALDH^+^ lung CSCs, where its knockdown reduces the expression of stemness-related markers and significantly impairs both colony formation and migratory capacity [27]. The Wnt/β-catenin pathway may contribute to the stemness of cancer cells [88]. Plectin activates the canonical Wnt/β-catenin signaling pathway by interacting with dishevelled-2, forming a stabilizing complex that protects dishevelled-2 from ubiquitin-mediated degradation [89]. Additionally, plectin knockdown has been shown to impair colony-forming potential in SL-13R-induced hematopoietic progenitor cells, suggesting a broader role in stem cell maintenance [90]. However, further investigation is needed to fully elucidate plectin’s role in CSC biology.

The contribution of plectin to drug resistance is also closely linked to its role in sustaining CSCs’ properties. Recent evidence has identified plectin as a key gene linked to the hypoxic TME in CRC, which is strongly correlated with therapeutic resistance [33]. In GBM, plectin was found to be upregulated in cisplatin-resistant cell lines, suggesting its role as a mediator of chemoresistance [91]. Plectin has also been implicated in resistance to histone deacetylase inhibitors (vorinostat, panobinostat) [43], topoisomerase II inhibitors (irinotecan), and microtubule-targeting agents (paclitaxel and vincristine) [92]. These findings collectively underscore the potential of plectin as a therapeutic target for overcoming drug resistance in multiple cancer types.

### 3.4. Bidirectional Regulation Between Plectin and TME

**Figure 2:**
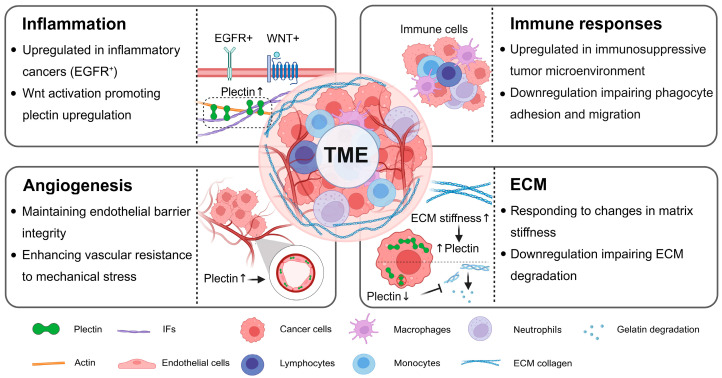
Bidirectional interactions between plectin and the TME. Plectin dysregulation contributes to TME remodeling, while TME-derived cues reciprocally induce plectin dysregulation. Specifically, plectin expression is upregulated (indicated by upward arrows) in EGFR^+^ inflammatory breast cancer and during Wnt-activated inflammatory signaling. Moreover, its upregulation is also observed in immunosuppressive tumor microenvironments. The loss of transmembrane protein 268, an upstream regulator of plectin, has been shown to influence phagocyte adhesion and migration. In addition, elevated plectin (indicated by upward arrows) further promotes the maintenance of vascular barriers, enhancing endothelial resistance to diverse mechanical stimuli and supporting angiogenesis within the TME. Finally, plectin upregulation (indicated by upward arrow preceding plectin) responds to increased ECM stiffness (indicated by upward arrow following ECM stiffness) to facilitate cancer cell invasion, whereas loss of plectin function (indicated by downward arrow following plectin) impairs gelatin degradation. Image created with BioRender.com.

The TME comprises diverse cellular and acellular components that critically influence tumor growth, invasion, metastasis, and cancer therapy. Emerging evidence indicates that dysregulation of plectin plays a role in both the regulation and response to the TME. Specifically, plectin is upregulated in pro-inflammatory tumors and immunosuppressive microenvironments. Its upregulation enhances angiogenesis and vascular integrity, and mediates ECM mechanotransduction and gelatin degradation. These findings highlight the bidirectional role of plectin in the TME, where it drives remodeling of the TME and is dysregulated due to TME alterations (As shown in Figure 2).

Inflammation is a hallmark of cancer, profoundly affecting tumor progression, malignant transformation, and therapeutic efficacy. Epidermal growth factor receptor (EGFR) serves as a key regulator in orchestrating the balance between immunosuppressive and immunostimulatory responses within the TME, particularly in inflammatory breast cancer [93]. Notably, plectin is significantly upregulated in inflammatory breast cancer cells exhibiting EGFR^+^ expression, where it modulates cell migration through the integrin/EGFR signaling axis [35]. The Wnt signaling pathway is another well-established mediator of inflammatory signaling [94], which also influences plectin expression. In human induced pluripotent stem cell (hiPSC)-derived organoids treated with CHIR99021—a Wnt pathway agonist—plectin levels were elevated relative to controls [95]. When exposed to Aβ42 oligomers, these organoids exhibited a heightened inflammatory response, as indicated by upregulated TNFα, IL-6, and MMP2/9 expression. Insights from hiPSC provide a potential framework for understanding tumor inflammation, although tumor-specific microenvironmental and genetic factors must also be considered.

Plectin dysregulation also contributes to the modulation of the tumor immune microenvironment. Machine learning-based analyses have identified plectin as a biomarker and a potential therapeutic target in immunologically cold pancreatic adenocarcinomas [34]. Higher *PLEC* gene expression is generally associated with an immunosuppressive tumor microenvironment enriched in M2 macrophages, while in “cold-high” tumors, *PLEC* gene expression is negatively correlated with M2 abundance, suggesting that it may indirectly inhibit M2 recruitment or function that remains to be elucidated [34]. In Glutathione-s-transferases theta two wild-type mice, plectin expression correlates with the enrichment of M2-type macrophages, suggesting a role in promoting an immunosuppressive microenvironment. Moreover, following bacillus calmette guerin treatment, increased plectin levels were associated with elevated PD-L1 expression in an immune-activated tumor context, further supporting its role in immune modulation [69,96]. Mechanistically, the Integrin β4/plectin signaling axis is regulated by transmembrane protein 268, and its disruption has been shown to impair phagocyte adhesion and migration, thereby influencing immune cell dynamics within the TME [49]. However, whether plectin downregulation directly affects macrophage infiltration remains to be experimentally validated.

Tumor angiogenesis is essential for sustaining tumor growth by providing oxygen and nutrients while facilitating the removal of metabolic waste [97]. The integrin β4/plectin/vimentin complex forms critical transmembrane linkages between vascular endothelial cells and the basement membrane, thereby contributing to endothelial barrier integrity [98]. Studies have shown that endothelial cell layers deficient in plectin exhibit increased permeability and diminished resistance to shear stress and mechanical strain, underscoring its role in vascular stability [99]. Furthermore, the knockdown of Notch2 in vascular smooth muscle cells results in reduced plectin expression during vascular remodeling, further highlighting its importance in maintaining vascular wall architecture [100]. Plectin is also vital for the formation and mechanical adaptation of vascular endothelial cells, particularly within the mechanically dynamic TME. Notably, plectin expression is upregulated in endothelial cells exposed to cyclic mechanical stretch, where it acts as a key mechanosensitive regulator [36]. This upregulation is accompanied by increased expression of vascular development-related genes, including ANGPTL4, ANGPTL5, and PDE1A, suggesting that plectin contributes to cytoskeletal remodeling and mechanotransduction in angiogenic endothelial cells.

The mechanical properties of the ECM critically influence tumor growth and metastasis by shaping the behavior of cancer cells within the TME [101]. Emerging evidence implicates plectin as a pivotal mediator of mechanotransduction—the process by which mechanical signals from the ECM are converted into intracellular responses. Plectin expression is modulated by ECM stiffness and correlates significantly with clinical prognosis in ovarian cancer patients [59]. Recent findings have shown that increased ECM stiffness activates the integrin β1–plectin–F-actin signaling axis, thereby enhancing the migratory capacity of HCC cells [32]. Conversely, silencing plectin impairs matrix-degrading capacity and reduces cytoskeletal remodeling in HCC cells [26]. These observations underscore the essential role of plectin in enabling cancer cells to sense and respond to biomechanical cues from the ECM, ultimately facilitating tumor progression through cytoskeletal stabilization and enhanced invasiveness.

## 4. Targeting Plectin Dysregulation in Cancer Therapy

Therapeutic strategies aimed at disrupting plectin dysregulation in cancer have demonstrated potential in suppressing tumorigenesis and progression, including gene knockout, RNA interference-mediated knockdown, and functional inhibition.

One particularly promising compound, PST—a ruthenium (arene) pyridinecarbothioamide complex—selectively targets plectin and inhibits its cytoskeletal crosslinking function. CRISPR/Cas9-based gene knockout of the *PLEC* gene reorganizes keratin networks by disrupting circumferential rims and radial spokes, increasing actin stress fibers, and enhancing actomyosin contractility, leading to elevated tension at adherens junctions and desmosomes [102]. These findings highlight plectin’s essential role in maintaining cytoskeletal integrity and tension homeostasis. Similarly, treatment with 16 μM PST for 4 h in MDCK cells, cholangiocytes, and MCF-7 cells mimics the plectin gene knockout phenotype, inducing keratin bundling, loss of the circumferential keratin rim, and desmosome widening and tortuosity. PST also increases cellular contractility and line tension at adherens junctions and desmosomes, as measured by traction force microscopy and FRET-based tension sensors. Inhibition of actomyosin activity with blebbistatin or Rho kinase inhibitors reverses these structural changes, confirming that PST-induced cytoskeletal remodeling is actomyosin-dependent.

Further studies additionally support PST as a potential novel anticancer agent [103,104,105]. PST inhibits plectin expression and disrupts the formation of the plectin–MT1-MMP complex, thereby inhibiting the formation of invasive pseudopodia and suppressing matrix degradation and cellular invasion [106]. Preclinical studies indicate that PST is generally well tolerated and effectively suppresses HCC progression in both cellular and murine models, with treatment typically applied at 8 μM (corresponding to 25% of the IC50 in Huh7 cells) and incubation times adjusted according to specific experimental assays [26]. These results provide mechanistic evidence that targeted inhibition of plectin disrupts cytoskeletal networks and contractility pathways, suppressing tumorigenic phenotypes and highlighting its therapeutic potential.

In addition to targeting plectin at the protein level, therapeutic strategies directed at its mRNA also represent a promising avenue. mRNA-based interventions have demonstrated efficacy in modulating plectin expression. For instance, miR-124-3p directly targets the 3′-UTR of *PLEC* mRNA, leading to its downregulation and thereby reversing drug resistance-associated EMT and invasive phenotypes in cancer cells [91]. Likewise, the virus-encoded artificial microRNA amiR-4 reduces plectin expression and enhances the cytotoxicity of cancer cells, highlighting the potential of RNA-based therapeutics in oncology [107].

However, most cytoskeleton-targeting chemotherapeutic agents act on actin or microtubules, leading to significant side effects due to the ubiquitous presence of these structural proteins in normal cells. In contrast, CSP is characterized by aberrant overexpression and mislocalization to the cell surface, providing a more selective and tumor-restricted therapeutic target. Monoclonal antibodies developed against CSP have demonstrated potent anticancer activity in preclinical studies [25]. Among these, ZB131 (developed by ZielBio) is currently undergoing phase 1–2 clinical trials and has shown good tolerability in adult patients with PDAC, cholangiocarcinoma, and ovarian cancer [108]. In addition, surface-targeting peptides that bind to plectin, such as a peptide derived from Cyclin D2 (Pep5) [40] and a highly CSC-specific hit peptoid (PSC2) [52], exhibit high binding specificity and have been shown to inhibit tumor growth or induce cancer cell death. Although growing evidence supports plectin as a promising therapeutic target, these inhibitors still lack sufficient clinical trial validation to confirm their efficacy and safety in clinical applications.

## 5. Conclusions and Perspectives

Plectin dysregulation is observed in many cancer types and is primarily characterized by abnormal expression and mislocalization of plectin. Its dysregulation can promote tumor growth and metastasis and contribute to the establishment of a tumor-supportive microenvironment. Therefore, inhibitors targeting plectin dysregulation have potential in cancer therapy. Although we have valuable clues that plectin dysfunction can promote cancer development, many areas still need attention regarding its dysregulation and effects.

Plectin dysregulation exhibits tumor heterogeneity, with both upregulation and downregulation observed even within the same cancer type, reflecting its dual roles in tumor progression. Its upregulation promotes proliferation, invasion, and metastasis by stabilizing cytoskeletal architecture and activating oncogenic signaling pathways. Meanwhile, downregulation of plectin can enhance motility and dissemination by weakening intercellular adhesion and cytoskeletal integrity, as observed in HCC and high-grade ovarian tumors [66]. Plectin expression is also modulated by TME-derived cues, which in turn remodel the TME and foster tumor niches. Although plectin upregulation has been validated in pancreatic, ovarian, and liver cancers with ample clinical samples, clinical evidence in other tumor types remains limited, and the distinct implications of up- versus downregulation are not yet fully understood. Recognizing plectin dysregulation is critical for clinical practice, as it may refine prognostic stratification and guide the development of personalized therapeutic strategies targeting plectin-associated pathways.

This heterogeneity may in part result from isoform-specific functions of plectin, which have not yet been fully elucidated. Alternative splicing of the *PLEC* gene generates isoforms with distinct N-terminal sequences, conferring precise control over cytoskeletal architecture, organelle positioning, and tissue-specific functions [14]. In cancer cells, certain isoforms are aberrantly localized to the cell membrane and appear to drive tumor-promoting processes through isoform-specific mechanisms. These isoform-specific functions may partly explain the tumor heterogeneity observed in plectin dysregulation. However, the widespread use of pan-plectin antibodies has limited the ability to dissect these functional differences, resulting in the underappreciation of isoform-specific contributions in cancer research. The development and application of isoform-specific antibodies are therefore crucial for delineating the divergent roles of plectin isoforms in tumor initiation and progression.

In addition, more comprehensive and cost-efficient methods for detecting plectin dysregulation are needed to provide robust evidence. Current approaches for assessing plectin expression include protein- and mRNA-level assays, transcriptomic analyses, and immunofluorescence and immunohistochemical staining of clinical samples. Its subcellular localization has also been investigated through methods such as immunohistochemistry, phage display, unbiased peptoid screening, and immunogold staining [23,27]. These strategies have already underscored the potential of plectin to be a possible biomarker in diverse cancers—for instance, its upregulation in melanoma metastasis, enrichment in the immune-cold microenvironment of pancreatic cancer [34], aberrant membrane localization as a marker of invasion [24], and involvement in stemness maintenance [27]. Its dysregulation has also been linked to poor prognosis in multiple cancers, underscoring its clinical relevance. Developing practical and clinically applicable detection platforms will facilitate broader screening and reinforce plectin’s promise as both a diagnostic and therapeutic target.

Notably, CSP exhibits aberrant localization that contributes to tumor stemness and metastasis. CSP has been identified as a surface marker for lung cancer stem cells, and its downregulation reduces the expression of stemness-associated genes, implying a potential role for plectin in maintaining stemness [27]. Plectin undergoes abnormal membrane localization through the exosome pathway in PDAC and affects tumor invasion and metastasis [24]. Moreover, CSP’s selective membrane expression in cancer cells offers a promising strategy for targeted drug delivery, enabling precise therapeutic delivery to tumor sites [23,109]. These findings underscore the protumorigenic functions of CSP that extend beyond its utility as a diagnostic biomarker. Building on advances in understanding plectin/CSP biology, the development of plectin/CSP-targeting therapies offers a promising avenue for improving cancer diagnosis, treatment, and prognosis.

## Figures and Tables

**Table 1:** Dysregulation of plectin in cancer.

Dysregulation	Cancer Types	Outcomes	References
Upregulation	AML	*PLEC* gene mutation	[37]
	Breast cancer	Promoting growth and metastasis; substrate for caspases; orchestrating PLEC/NFκB1/CXCL9 axis; SNRPA1-mediated *PLEC* alternative splicing	[38,39,40,41,42]
	Bladder cancer	Promoting invasion and metastasis; promoting invadopodia formation	[43,44]
	CRC	Promoting invasion; targeting podosome-like adhesions; contributing to drug resistance	[18,33]
	ESCC	Maintaining anchorage and proliferation; increasing ESCC risk	[45,46,47]
	GBM	Enhancing migration; regulating morphological changes	[48]
	Gastric cancer	Promoting growth; integrin β4–plectin complex forming	[49]
	HCC	Promoting migration and invasion; promoting EMT; involving the Integrin β1/Plectin/F-actin axis; upregulating in high matrix stiffness	[26,31,32]
	HNSCC	Promoting migration and invasion; upregulating Erk 1/2 kinase	[50]
	Lung cancer	Enhancing invasion and migration; maintaining stemness	[27,51,52]
	Melanoma	Metastasis biomarker; promoting growth; activating Src signaling	[29,53,54,55]
	OSCC	Prognostic marker; enhancing cell motility, invasion, and tumorigenicity	[8,28]
	Ovarian cancer	Promoting migration, invasion, and adhesion; upregulating in high substrate stiffness	[56,57,58,59]
	Pancreatic cancer	Malignant biomarker; promoting proliferation, migration, and invasion; participating in integrin β4-dependent exosomal transport	[23,24,60]
	Prostate cancer	Promoting growth and metastasis; targeting focal adhesions	[30,61,62]
	Testicular cancer	Novel susceptibility genes	[63]
Downregulation	BCC and SCC	Promoting invasion	[64]
	ESCC	Disrupting stratified squamous epithelium homeostasis	[45]
	HCC	Promoting cell motility; activating FAK and Rac1-GTPase	[65,66,67]
	Ovarian cancer	Downregulating during tumor progression; promoting EMT	[56]
Mislocalization	CRC	Plectin 1k targeting podosome-like adhesions	[18]
	ESCC	Localizing in the cell membrane	[47]
	GBM	Co-localization with membrane-related-aquaporin 4 aggregates	[48]
	HCC	Perimembranous enrichment	[26]
	HNSCC	Uniform in the cytoplasm and the cell membrane	[50]
	Lung cancer	Biomarker of ALDH^+^ lung cancer stem cells	[27]
	OSCC	Mainly at the plasma membrane	[28]
	Ovarian cancer	Monoclonal antibodies targeting CSP; a target for drug delivery	[56,57,58]
	Pancreatic cancer	Participating in exosome formation and enhancing tumor growth	[23,24]

ALDH: Aldehyde dehydrogenase; AML: Acute myeloid leukemia; BCC and SCC: Basal and squamous cell carcinomas; CRC: Colorectal cancer; CSP: cancer-specific plectin; EMT: Epithelial–mesenchymal transition; Erk 1/2: Extracellular signal-regulated kinase 1/2; ESCC: Esophageal squamous cell carcinoma; FAK: focal adhesion kinase; GBM: Glioblastoma multiforme; HCC: Hepatocellular carcinoma; HNSCC: Head and neck squamous cell carcinoma; OSCC: Oral squamous cell carcinoma; Rac1: Ras-related C3 botulinum toxin substrate 1; Src: Rous sarcoma oncogene.

## Data Availability

No new data were created or analyzed in this study.

## Abbreviations

The abbreviations listed below are used in this manuscript:

AFs	Actin filaments
ABD	Actin-binding domain
AML	Acute myeloid leukemia
ALDH	Aldehyde dehydrogenase
BCC	Basal cell carcinomas
CSCs	Cancer stem cells
CSP	Cancer-specific plectin
CRC	Colorectal cancer
ECM	Extracellular matrix
EGFR	Epidermal growth factor receptor
EMT	Epithelial–mesenchymal transition
Erk 1/2	Extracellular signal-regulated kinase 1/2
ESCC	Esophageal squamous cell carcinoma
FAs	Focal adhesions
FAK	Focal adhesion kinase
GBM	Glioblastoma multiforme
HNSCC	Head and neck squamous cell carcinoma
HDs	Hemidesmosomes
HCC	Hepatocellular carcinoma
IFs	Intermediate filaments
OSCC	Oral squamous cell carcinoma
PDAC	Pancreatic ductal adenocarcinoma
PanINs	Pancreatic intraepithelial neoplasms
PST	Plecstatin-1
SCC	Squamous cell carcinomas
Src	Rous sarcoma
SSE	Stratified squamous epithelium
Rac1	Ras-related C3 botulinum toxin substrate 1
TME	Tumor microenvironment
